# Soluble lymphocyte activation gene-3 (sLAG3) and CD4/CD8 ratio dynamics as predictive biomarkers in patients undergoing immune checkpoint blockade for solid malignancies

**DOI:** 10.1038/s41416-023-02558-7

**Published:** 2024-01-17

**Authors:** Joao Gorgulho, Christoph Roderburg, Fabian Beier, Carsten Bokemeyer, Tim H. Brümmendorf, Sven H. Loosen, Tom Luedde

**Affiliations:** 1 Department of Oncology, Hematology and Bone Marrow Transplantation with Section of Pneumology, University Medical Centre Hamburg-Eppendorf, Martinistraße 52, 20251 Hamburg, Germany; 2 Mildred Scheel Cancer Career Center, University Cancer Center Hamburg, University Medical Center Hamburg-Eppendorf, Hamburg, Germany; 3 Department of Gastroenterology, Hepatology and Infectious Diseases, University Hospital Düsseldorf, Medical Faculty of Heinrich Heine University Düsseldorf, 40225 Düsseldorf, Germany; 4 Center for Integrated Oncology Aachen-Bonn-Cologne-Düsseldorf (CIOABCD), Aachen, Germany; 5 Department of Medicine IV, University Hospital RWTH Aachen, Pauwelsstrasse 30, 52074 Aachen, Germany

**Keywords:** Tumour biomarkers, Prognostic markers, Tumour biomarkers

## Abstract

**Background:**

The search for biomarkers to identify suitable candidates for immune checkpoint inhibitor (ICI) therapy remains ongoing. We evaluate how soluble levels of the next generation immune checkpoint Lymphocyte Activation Gene-3 (sLAG-3) and its association with circulating T lymphocyte subsets could pose as a novel biomarker to predict outcome to ICI therapy.

**Methods:**

Circulating levels of sLAG3 were analyzed using multiplex immunoassay in *n* = 84 patients undergoing ICI therapy for advanced solid cancer, accompanied by flow cytometry analyses of peripheral blood mononuclear cells (PBMCs).

**Results:**

Uni- and multivariate analysis shows that patients with higher sLAG3 concentrations before ICI therapy had a significantly impaired progression-free (PFS) and overall survival (OS) (HR_PFS_: 1.005 [95%CI: 1.000–1.009], *p* = 0.039; HR_OS_: 1.006 [95%CI: 1.001–1.011], *p* = 0.015). The CD4/CD8 cell ratio and its dynamics during therapy were strong predictors of PFS and OS with patients with a decreasing ratio between baseline and after 1–2 cycles having an improved median OS compared to patients with increasing values (*p* = 0.012, HR: 3.32). An immunological score combining sLAG3 and the CD4/CD8 ratio showed the highest predictive potential (HR_OS_: 10.3).

**Conclusion:**

Pending prospective validation, sLAG3 and correlating circulating T-cell subsets can be used as a non-invasive predictive marker to predict outcome to ICI therapy to help identifying ideal ICI candidates in the future.

## Background

Antibodies towards programmed cell death protein and its ligand (PD-1/PD-L1) as well as cytotoxic T-lymphocyte activator-4 (CTLA-4), that boost the host’s immune system to combat a malignancy, revolutionized the landscape of cancer therapy and have been approved for a wide range of malignancies [1–3]. Currently, durable responses to ICI therapy are limited to less than one third of patients. The need to find minimally invasive, feasible biomarkers that reliably aid physicians in ideal patient selection is ongoing with many already identified, being PD-L1 tumor scoring and the tumor mutational burden (TMB) the most established in clinical use, with some limitations [4]. The lymphocyte activation gene-3 (LAG3) is a coinhibitory receptor expressed by a wide range of immune cells, mainly T lymphocytes [5]. Its main and mostly understood ligand is the major histocompatibility complex II (MHC-II), expressed by professional antigen presenting cells (APCs) with at least four others identified: fibrinogen like protein-1 (FGL-1), galectin-3 (Gal-3), lymph node sinusoidal endothelial cell C-type lectin (LSECtin) and α-synuclein fibrils (α-syn). Interactions of LAG3 with its ligands ultimately lead to negative regulation of T cell activity [6, 7]. Its soluble form, sLAG3, produced via alternative splicing and proteolytic cleavage of the extracellular domain, is mainly secreted by dendritic cells (DCs) but also B and T lymphocytes and its function remains mainly controversial and unknown. Some studies show its role as a costimulatory molecule by binding to MHC-II and promoting the maturation of DCs, these are however mostly related to synthetic sLAG3-Ig proteins used in a therapeutic fashion, which possess a high MHC-II affinity, contrarily to human sLAG3 [8, 9]. However, human sLAG3 has been suggested to act as a co-inhibitor of immune responses by impairing monocyte differentiation and antigen presenting capability preventing their induction of T cell proliferation [10, 11]. We hypothesize therefore that sLAG3 acts as a coinhibitory immune modulator impeding an effective antitumoral immune response for patients before and during immune checkpoint blockade. The diagnostic and prognostic potential of this soluble molecule has been studied outside the cancer spectrum in the context of infection [12] and Parkinson’s disease [13], in different tumors outside the immunotherapy spectrum [14, 15], but within the frame of ICI based therapy it has been scarcely exploited, with one study in head and neck cancer showing how high pretreatment serum levels of sLAG-3 predicted worsened PFS and OS [16].

In this basket trial, we aim to address this issue, evaluating the predictive role of serum sLAG3 levels before and throughout therapy and their association with the expression of different circulating T-cell subsets.

## Patients and methods

### Study population

This cohort study aimed to evaluate the predictive role of circulating sLAG3 levels, by assessing the relation between serum concentrations of this molecule, tumor response, toxicity and outcome in patients undergoing therapy with ICIs for different advanced solid cancers. In a further step, these concentrations were matched with frequency of different T cell subsets in the peripheral blood using flow cytometry. From August 2017 to September 2019, we prospectively recruited *n* = 84 patients at the interdisciplinary cancer outpatient clinic at the University Hospital RWTH Aachen with advanced stage disease (UICC III or IV) (see Table 1 for patient characteristics) who were planned to be treated with an ICI. Both patients who progressed to their prior systemic therapy (no limit of prior lines), as well as patients being treated with an ICI in a first line-setting were included. The ethics committee of the University Hospital RWTH Aachen, Germany (EK 206/09) approved the study protocol, which was conducted in accordance with the ethical standards laid down in the 1964 Declaration of Helsinki and its later amendments. Before recruitment, all patients delivered written informed consent. Blood samples were collected prior to ICI therapy and throughout treatment at two different time points (early time point: after one to two cycles of therapy, late time point: after three to five cycles). By centrifugating the whole blood samples for 10 min at 2000 × *g* we isolated serum samples and stored them at −80 °C until use. For flow cytometry-based analyses of the immune status, one EDTA tube per patient (*n* = 63) was drawn and freshly prepared. In addition, for flow cytometry assessing LAG3 expression (*n* = 6), peripheral blood mononuclear cells (PBMCs) were isolated from whole blood by using the BD Vacutainer® CPT™ System (Cat No.:362782, BD Bioscience, USA), following manufacturer’s instructions [17] and cryopreserved with 10% DMSO at –80 °C. As a control population, blood was drawn and serum was isolated from a total of *n* = 32 healthy, cancer-free blood donors. The histological tumor diagnosis was performed by experienced pathologists, as well as the PDL-1 immunohistochemical staining and scoring, performed at the diagnosis timepoint, or later upon request of the treating oncologist. The evaluation of tumor response was based on clinical and radiological evaluation and patients were stratified into three groups: responders (R): including patients with complete response (CR) and partial response (PR), stable (S): including patients with stable disease (SD) and non-responders (NR), who exhibited progressive disease (PD). Clinical endpoints chosen were progression-free survival (PFS) and overall survival (OS), which were evaluated in October 2020, with the first patient being recruited in August 2017 and the last in May 2019.

**Table 1:** Patient characteristics.

Parameter	Study cohort
Cancer patients	*n* = 84
Gender [%]:	
male-female	64.2–35.7
Age [years, median and range]	67.5 [38–87]
BMI [kg/m^2^, median and range]	24.1 [15.9–42.3]
Tumor entity (prior / no prior therapy) [%]	
NSCLC	40.5 (41.7 / 37.5)
Melanoma	13.1 (8.3 / 25.0)
Urogenital tract	13.1 (11.7 / 16.7)
GIT	14.3 (16.7 / 8.3)
Head and neck	10.7 (15.0 / 0.0)
Other malignancies	8.3 (6.7 / 12.5)
Staging [%]	
UICC III	6.2
UICC IV	93.8
ECOG PS [%]	
ECOG 0	7.1
ECOG 1	51.2
ECOG 2	39.3
ECOG 3	2.3
Therapeutic agent [%]	
Nivolumab	59.5
Pembrolizumab	28.5
Nivolumab/Ipilimumab	6.0
Other (Avelumab, Durvalumab)	6.0
Smoker status [%]	
Never	9.5
Yes, ex	39.2
Yes, present	22.6
Unknown	28.5
Prior lines of systemic therapy [%]	
0	28.6
1	50.0
2	15.5
3 or more	6.0
Side effects [%]	
Any	39.3
CTC G3 or higher	11.9
PDL-1 score (prior / no prior therapy) [%]	
≥50%	21.5 (15.0/37.6)
10–49%	6.0 (6.7/4.2)
1–10%	8.4 (8.4/8.4)
0%	25.0 (31.7/8.3)
unknown	39.3 (38.3/41.7)

*BMI* body mass index, *ECOG PS* “Eastern Cooperative Oncology Group” performance status, *NSCLC* non-small cell lung cancer, *GIT* gastrointestinal tract, *CTC* common toxicity criteria.

### Evaluation of sLAG3 serum levels

Serum concentrations of sLAG3 were measured by multiplex immunoassay in accordance with the manufacturer’s instruction using a Bio-Plex 200 system and Bio-Plex Manager 6.0 software (Immuno-Oncology Checkpoint 14-Plex Human ProcartaPlex™ Panel 1, #EPX14A-15803-901, ThermoFischer Scientific, USA).

### Evaluation of frequency of T cell subsets in peripheral lymphocytes by multicolor flow cytometry

In a subset of patients (*n* = 63), freshly isolated cells were lysed using the Immunoprep Reagent System (Beckman Coulter) and stained with CD45-FITC/CD56-PE/CD19-ECD/CD3-PC5 and CD45-FITC/CD4-PE/CD8-ECD/CD3-PC5 antibody mixes (all Tetrachrome, Beckman Coulter, Krefeld, Germany), according to manufacturer´s instructions. Flow-cytometry analysis was carried out and analyzed using NAVIOS cytometer and analysis software (Beckman Coulter).

In a small patient subpopulation, from which pretreatment PBMCs were available (*n* = 6, patient characteristics in Supplementary Table 1), expression of membrane bound LAG3 was quantified in T lymphocytes, key players of antitumoral response. For this instance, priorly isolated and cryopreserved PBMCs were thawed and stained for flow cytometry analysis using fluorescently labeled mAbs directed towards CD3, CD4, CD8 and LAG3, in order to quantify LAG3 positive Helper T lymphocytes (HTLs) (CD3 + CD4 + CD8-LAG3 + ) and cytotoxic T lymphocytes (CTLs) (CD3 + CD4-CD8 + LAG3 + ) (CD3: Clone UCHT1, Fluorophor APC-R700 ; CD4: Clone SK3, Fluorophor BUV737 ; CD8: Clone RPA-T8, Fluorophor BV786; LAG3: Clone T47-530, Fluorophor Alexa 647; all originating from BD Biosciences, San Jose, CA, USA). A LSR Fortessa (BD Biosciences, San Jose, CA, USA) was used for cytometry analysis and FlowJo 10.6 software (TreeStar, Ashland, OR, USA) for data analyses.

### Statistical analysis

With the Shapiro-Wilk-Test, we checked for normal distribution of the data. Non-parametric data were compared by the Mann–Whitney-U-Test and Kruskal-Wallis-H-Test. To demonstrate the median, quartiles and ranges box plot graphics were used. ROC curves were generated by plotting the sensitivity against 1-specificity. Kaplan-Meier curves aided in showing the influence of a specific parameter on overall survival (OS) and progression-free survival (PFS), and the Log-rank test was applied to analyze statistical differences between subgroups. For a longitudinal comparison of differences in sLAG-3 serum levels between the three time-points (before ICI treatment, early time-point, and late time-point), we applied repeated measures ANOVA. The main F-test is reported. For calculation of the ideal cut-off serum concentration of sLAG3 and CD4/CD8 ratio in regard to OS we employed the “Charité cut-off finder”. In order to show the robustness of this OS related cut-off, the same cut-off was employed for PFS analyses. This is a publicly available software-tool, which fits Cox proportional hazard models to the dichotomized survival status (deceased or alive) as well as the survival time (duration between first ICI administration and death/last follow-up) and defines the optimal cut-off as the sLAG3 concentration or CD4/CD8 ratio with the most significant split in log-rank test [18]. To corroborate the predictive value of variables, uni- and multivariate Cox-regression were performed. Parameters with a *p* value of <0.100 in univariate testing were included into multivariate analysis. The hazard ratio (HR) and the 95% confidence interval are displayed. The Spearman correlation coefficient was used for correlation analyses between flow cytometry data and serum concentrations of sLAG3. All statistical analyses were performed using SPSS 23 and 25 (SPSS, Chicago, IL, USA). A *p* value of <0.05 was considered statistically significant (*p < 0.05; **p < 0.01; ***p < 0.001).

## Results

### Study population

The study population was composed of *n* = 84 patients with advanced cancer (UICC III/IV) treated with ICIs (Table 1). Patients had a median age of 67.5 years (range: 38–87 years) with 64.2% and 35.8% being male and female, respectively. The most frequent tumor entity was non-small cell lung cancer (NSCLC) (40.5%), followed by malignant melanoma (13.1%), urogenital cancer (13.1%), gastrointestinal (GI)-cancers (14.2%), head and neck tumors (10.7%) and others (8.4%), most of which were metastasized before start of ICI therapy (93.8% in UICC stage IV). Concerning toxicity, 39.3% of patients exhibited immune related adverse events (IRAE) of any grade. Toxicity graded 3 or higher was observed in 11.9% of patients. Patients who received therapy with ICI as a first-line systemic treatment (28.6%) were mainly chosen based on their PDL-1 score (mainly NSCLC with a PDL-1 expression ≥50% (37.5%)) and/or tumor entity (mainly malignant melanoma (25%), urogenital tract (16.7%) and other malignancies, all of these being merkel cell carcinomas (12.5%)).

### Baseline sLAG3 concentrations in cancer patients

**Fig. 1:**
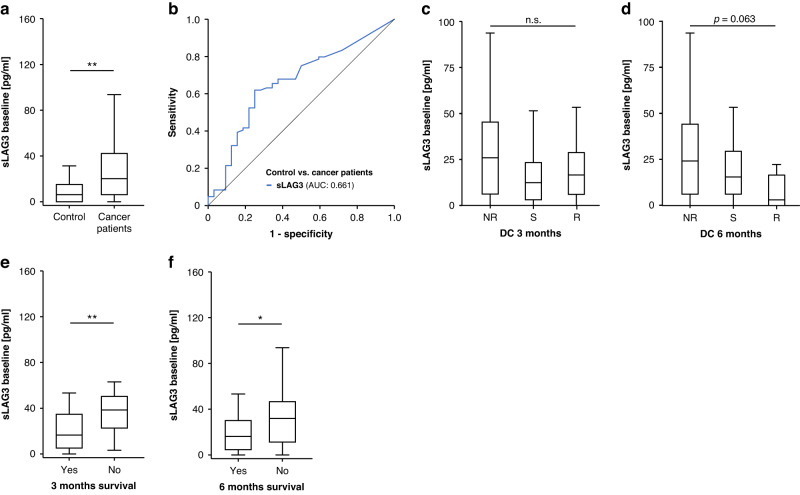
sLAG3 serum concentrations are significantly elevated in cancer patients and show a trend in response prediction to immune checkpoint inhibitor therapy while playing a clear role in prediction of overall survival. **a** sLAG3 serum concentrations are significantly higher in cancer patients compared to healthy controls. **b** ROC curve analysis reveals an AUC value of 0.661 for sLAG3, denoting the clear difference between levels in cancer patients compared to healthy controls. Serum concentrations of sLAG3 before therapy show a non-significant trend towards higher levels in non-responders (NR) to ICI therapy compared to patients with stable disease (S) and responders (R) at 3 (**c**) and 6 months (**d**). Patients who still live 3 months (**e**) and 6 months (**f**) after therapy initiation show significantly lower levels of sLAG3 in the peripheral blood (p_3month_ = 0.003, p_6month_ = 0.016).

In a first analysis, to assess the relevance of circulating sLAG3 concentrations in advanced cancer, we compared sLAG3 serum levels before therapy with those of healthy controls. These were significantly higher in cancer patients (median sLAG3: 20.10 vs. 6.06 pg/ml, *p* = 0.007 Fig. 1a). ROC curve analysis showed an AUC value of 0.661 for the differentiation between cancer patients and healthy controls (Fig. 1b). Serum sLAG3 concentrations were then compared between diverse clinicopathological categories including cancer entity, sex, tumor stadium (UICC), ECOG performance status, smoking status, chosen ICI regimen and line of therapy. Besides significantly higher circulating sLAG3 baseline values in metastatic disease (UICC stage IV, *p* = 0.036, Supplementary Fig. 1C), no significant differences could be seen among the different groups (Supp. Fig. 1a, b, d–g).

#### sLAG3 concentrations before therapy do not predict tumor response at a 3 and 6-month timepoint or toxicity, but predict overall survival at a 3 and 6 month timepoint

We then aimed to assess whether the three ICI therapy response groups (R with CR/PR, S with SD, NR with PD) could be discriminated based on circulating sLAG3 levels before therapy. Despite a trend towards higher sLAG3 levels at 3 and 6 months among non-responders (NR) compared to responders (R) and patients with stable disease (SD) this difference was non-significant (Fig. 1c, d). Regarding overall survival (OS), we used the 3- and 6-months threshold to categorize patients. Here, we observed significantly lower sLAG3 levels among patients who were still alive after 3 and 6 months, respectively (Fig. 1e, f). Moreover, sLAG3 serum concentrations did not significantly differ between patients with or without IRAE as well as patients experiencing IRAE grade 3 or higher (Supplementary Fig. 1H, I).

### Circulating levels of sLAG3 before ICI therapy are an independent predictor of progression-free survival and overall survival

**Fig. 2:**
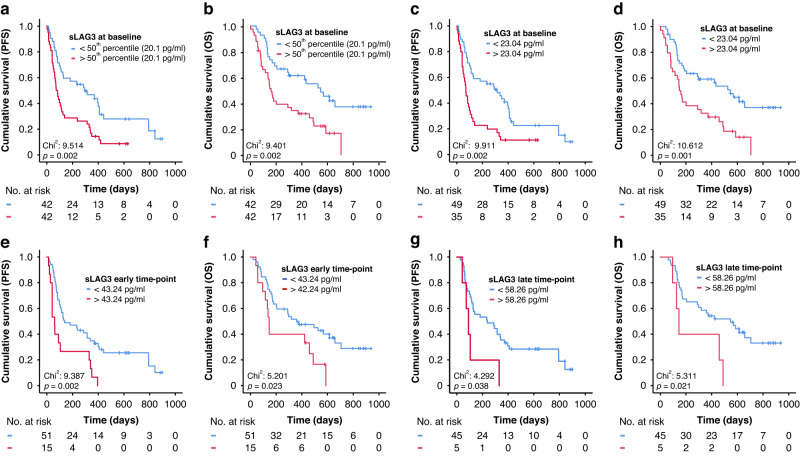
Baseline and longitudinal concentrations of sLAG3 predict progression-free and overall survival in patients undergoing immune checkpoint blockade. Using the median sLAG3 to split patients into two groups (20.1 pg/ml), patients with concentration above the median show strongly impaired PFS (**a**) and OS (**b**) compared to patients with levels below this cut-off. The predictive potential of sLAG3 levels before therapy is even higher when applying an ideal cut-off value (23.04 pg/ml), with patients with concentrations below the ideal cut-off having a median PFS of 314 days (**c**) and surviving in median 547 days (**d**) compared to a PFS of 71 and an OS of 141 days for patients with levels above the cut-off. **e**–**h** Patients with sLAG3 concentrations above the ideal cut-off value (early time point: 43.24 pg/ml, late time point: 58.26 pg/ml) show a significantly shorter PFS and OS compared to patients with lower circulating sLAG3 levels during the course of ICI treatment.

Observing the strong tendency towards higher sLAG3 levels before therapy in non-responder patients and the significantly higher sLAG3 levels in patients alive at 3- and 6 months after therapy commencement, we next looked at how sLAG3 could impact the progression-free survival (PFS) and overall survival (OS). For the purpose of Kaplan-Meier analysis, we first split patients at the median sLAG3 level (20.1 pg/ml) into two groups and could demonstrate a significant predictive relevance of sLAG3 levels regarding PFS (*p* = 0.002, Fig. 2a) and OS (*p* = 0.002, Fig. 2b). We then established an ideal predictive cut-off value for sLAG3 of 23.04 pg/ml based on the overall survival. Patients with sLAG3 concentrations above this cut-off had significantly impaired progression-free survival (median PFS: 314 days) and survived significantly shorter (median OS: 547 days) compared to patients with baseline sLAG3 levels below this threshold (median PFS 71 days, *p* = 0.002; median OS 141 days, *p* = 0.001, Fig. 2c, d). In order to further corroborate our findings, we employed uni- and multivariate Cox-regression analysis to exclude possible confounders. The initial univariate analysis further sustained our thesis confirming sLAG3 as a predictor of PFS (HR: 1.006 [95%CI: 1.002–1.011] *p* = 0.003, Table 2) and OS (HR: 1.004 [95%CI: 1.000–1.009] *p* = 0.039, Table 2). We then included several clinicopathological parameters of prognostic relevance (*p* < 0.110 in univariate analysis) into multivariate testing. Here, circulating sLAG3 concentrations demonstrated to be an independent predictor for PFS (HR: 1.005 [95%CI: 1.000–1.009], *p* = 0.039, Table 2) and OS (HR: 1.006 [95%CI: 1.001–1.011], *p* = 0.015, Table 2).

**Table 2:** Uni- and multivariate Cox-regression analysis for the prediction of progression-free and overall survival.

PFS	univariate Cox-regression		multivariate Cox-regression	
Parameter	*p* value	Hazard-ratio (95% CI)	*p* value	Hazard-ratio (95% CI)
sLAG3 baseline	0.003	1.006 (1.002–1.011)	0.039	1.005 (1.000–1.009)
Age	0.761	1.004 (0.979–1.030)		
Sex	0.599	0.877 (0.538–1.430)		
UICC tumor stage	0.263	1.940 (0.608–6.193)		
Tumor entity	<0.001	1.333 (1.160–1.532)	<0.001	1.310 (1.134–1.513)
Prior therapy	0.234	1.395 (0.806–2.416)		
ICI regimen	0.594	0.924 (0.691–1.235)		
ECOG PS	0.253	1.257 (0.849–1.861)		
Leukocyte count	0.264	1.033 (0.976–1.094)		
Sodium	0.107	0.947 (0.887–1.012)	0.164	0.949 (0.881–1.022)
Potassium	0.354	0.805 (0.509–1.273)		
ALT	0.179	1.004 (0.998–1.011)		
AST	0.270	1.005 (0.996–1.013)		
Bilirubin	0.101	1.527 (0.920–2.535)	0.451	1.213 (0.734–2.007)
Creatinine	0.814	0.814 (0.781–1.370)		
LDH	0.777	1.000 (0.998–1.002)		

OS	univariate Cox-regression		multivariate Cox-regression	
Parameter	*p* value	Hazard-ratio (95% CI)	*p* value	Hazard-ratio (95% CI)
sLAG3 baseline	0.039	1.004 (1.000–1.009)	0.015	1.006 (1.001–1.011)
Age	0.679	1.006 (0.979–1.033)		
Sex	0.877	0.871 (0.507–1.498)		
UICC tumor stage	0.450	1.726 (0.418–7.125)		
Tumor entity	<0.001	1.328 (1.138–1.550)	0.038	1.195 (1.010–1.414)
Prior therapy	0.363	1.325 (0.723–2.428)		
ICI regimen	0.553	0.907 (0.658–1.252)		
ECOG PS	0.030	1.613 (1.049–2.482)	0.002	2.119 (1.321–3.401)
Leukocyte count	0.184	1.038 (0.982–1.097)		
Sodium	0.133	0.953 (0.894–1.015)		
Potassium	0.837	0.946 (0.558–1.604)		
ALT	0.011	1.011 (1.002–1.019)	0.046	1.017 (1.000–1.033)
AST	0.055	1.010 (1.000–1.021)	0.864	0.997 (0.979–1.018)
Bilirubin	0.147	1.510 (0.865–2.635)		
Creatinine	0.449	0.852 (0.562–1.291)		
LDH	0.924	1.000 (0.998–1.002)		

*sLAG3* soluble lymphocyte activation gene-3, *UICC* Union for International Cancer Control, *AST* aspartate transaminase, *BMI* body-mass-index, *ECOG PS* Eastern Cooperative Oncology Group performance status, *LDH* lactate dehydrogenase

### Circulating levels of sLAG3 throughout ICI therapy predict progression-free survival and overall survival

In order to fill a relevant gap in soluble immune checkpoint and cytokine-based biomarker studies, we assessed the predictive potential of sLAG3 concentrations during ICI therapy. Two time points were considered: an early time point, after only one or two cycles of therapy (*n* = 67), to evaluate immediate immunotherapy effects and a late time point, after three to five cycles of therapy (*n* = 50), to evaluate a steadier immune state while on therapy. We compared sLAG3 levels at these timepoints with the respective baseline values but did not observe any significant changes (F (1.36, 59.92) = 2.040, *p* = 0.153, Supplementary Fig. 2A). We then proceeded as before, calculating ideal cut-off values of sLAG3 to discriminate long- and short-term responders and survivors (early time point: 43.24 pg/ml, which is also the value for the 75th percentile), late time point: (58.26 pg/ml). Patients with a sLAG3 concentration above the ideal cut-off had a significantly shortened PFS and OS for both time points during therapy (Fig. 2e–h). The predictive potential of sLAG3 values above the ideal cut-off for both time points was again confirmed by Cox-regression (for PFS: HR_early_: 2.509 [95%CI: 1.359-4.632], p = 0.003; HR_late_: 2.665 [95%CI:1.011-6.975], *p* = 0.047 ; for OS: HR_early_: 2.115 [95%CI: 1.094–4.091], *p* = 0.026; HR_late_: 2.992 [95%CI:1.123–7.7970], *p* = 0.028). Finally, we evaluated how decreasing or increasing sLAG3 concentrations between baseline and both time points impacted PFS and OS, where no significant differences could be seen (PFS not shown, OS in Supplementary Fig. 2B, C).

### Correlation between circulating T cell subsets and serum sLAG3 levels

In a next step, we assessed how sLAG3 concentrations were associated with key players of the antitumoral immune response and how their combined levels could predict the outcome of patients undergoing ICI therapy. We explored the correlation between sLAG3 and the abundance of several circulating T cell subsets in a patient subset (*n* = 63), such as T lymphocytes in total (CD3+), Helper T-cells (HTLs, CD4 +), cytotoxic T lymphocytes (CTLs, CD3 + CD8 +), as well as the CD4/CD8 cell ratio. Significant correlations could be shown between sLAG3 and CD8+ cells as well as between sLAG3 and the CD4/CD8 ratio (Supplementary Fig. 3A–C). In another patient subset (*n* = 6), we finally aimed at analyzing a potential correlation between sLAG3 and membrane-bound LAG3 on the surface of peripheral T lymphocytes and demonstrated a significant negative correlation between both. Supplementary Figure 4 depicts the different LAG3 expressions across all six patients on CTLs (Supplementary Fig. 4A–C) and HTLs (Supp. Fig. 5a, b). Interestingly, not only the frequencies of the cells correlated negatively with sLAG3 levels, but also their absolute counts (Supplementary Fig. 4D-E, 5C, D). More detailed values on laboratorial markers and cell populations are displayed in Supplementary Tables 2, 3.

### Predictive potential of CD4/CD8 ratio before and throughout ICI therapy

**Fig. 3:**
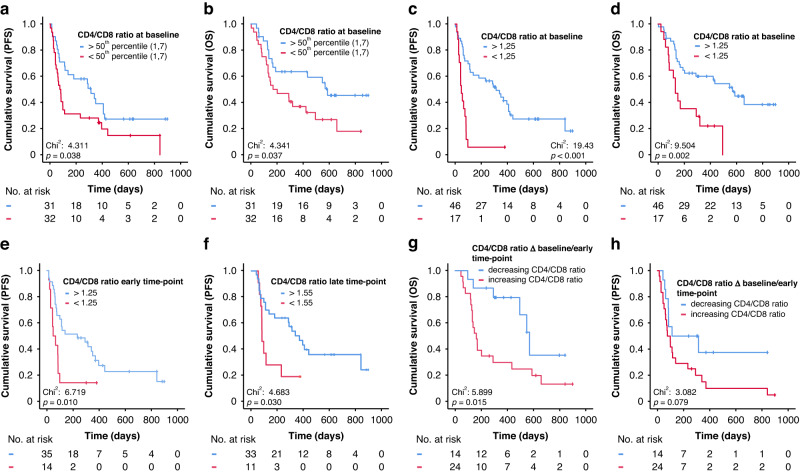
CD4/CD8 ratio values predict response-free and overall survival in patients undergoing ICI therapy before and during treatment. Progression-free and overall survival is significantly higher in patients with CD4/CD8 ratio values above the median of 1.7 (**a**, **b**) and even more above the ideal cut-off of 1.25 (**c**, **d**), than in those with values below these cut-offs. During therapy with ICI, at an early (**e**) and late time point (**f**), there is a significant difference in PFS between patients with values below or above respective ideal cut-offs (for early: 1.25, for late: 1.55). Rather than static values, dynamics of CD4/CD8 ratio during therapy (increasing/decreasing) are significant predictors of OS. Patients with decreasing CD4/CD8 ratio live significantly longer than patients with increasing values, when comparing early (**g**) values to the baseline. For PFS, only a non-significant trend can be depicted towards more durable response in patients with decreasing ratio (**h**).

In a further step, we took a closer look at the cell populations that correlated with sLAG3. In this case, we decided to focus on CD4/CD8 cell ratio, since it comprises both populations of T lymphocytes which are the main cellular players in the antitumoral immune response [19, 20]. Interestingly, in this cohort, the CD4/CD8 ratio showed relevance in predicting response as well as overall survival in patients undergoing ICI therapy. Kaplan-Meier analysis pointed CD4/CD8 ratio out as a significant predictor progression-free survival and overall survival. Patients with values over the median CD4/CD8 ratio (1.7) exhibited significantly longer progression-free (*p* = 0.038, Fig. 3a) and overall survival (*p* = 0.037, Fig. 3b). Again, an ideal cut-off (1.25) was calculated, which discriminated even more effectively between long- and short-term responders (*p* < 0.001, Fig. 3c) and survivors (*p* = 0.002, Fig. 3d). Cox regression confirmed values above the ideal CD4/CD8 ratio cut-off as a potent predictor of PFS and OS (for PFS: HR: 4.075 [95%CI: 2.081–7.983], *p* < 0.001; for OS: HR: 2.848 [95%CI: 1.420–5.712], *p* = 0.003). Longitudinal CD4/CD8 ratio values (n_early_=49, n_late_ = 44) during therapy were significantly different between patients with a longer and shorter PFS (cut-off_early_ = 1.25, p = 0.010; cut-off_late_ = 1.55, *p* = 0.030, Fig. 3e, f), while for OS only a strong trend could be observed (p_early_=0.062, p_late_ = 0.066) towards higher levels in patients who survived longer. Intriguingly, whilst static longitudinal values failed to predict OS, the individual dynamics of the CD4/CD8 ratio demonstrated a high predictive potential when looking at the differences between baseline and late time-point and especially between values before therapy and immediately after therapy initiation (*p* = 0.015 for ∆ baseline/early time-point, Fig. 3g; *p* = 0.047 for ∆ baseline/late time-point) with patients with a decreasing CD4/CD8 ratio surviving significantly longer compared to patients with an increasing CD4/CD8 ratio. Results for ∆ baseline/early time-point were confirmed in Cox-regression analysis (HR_early/baseline_: 3.324 [95%CI: 1.229–8.984], *p* = 0.018). For PFS, a not statistically significant tendency could be shown towards better response for patients with a decreasing CD4/8 ratio (*p* = 0.079 for ∆ baseline/early time-point, Fig. 3h).

### A combined immunological score comprising CD4/CD8 ratio dynamics and sLAG3 concentrations is the strongest predictor of progression-free and overall survival

**Fig. 4:**
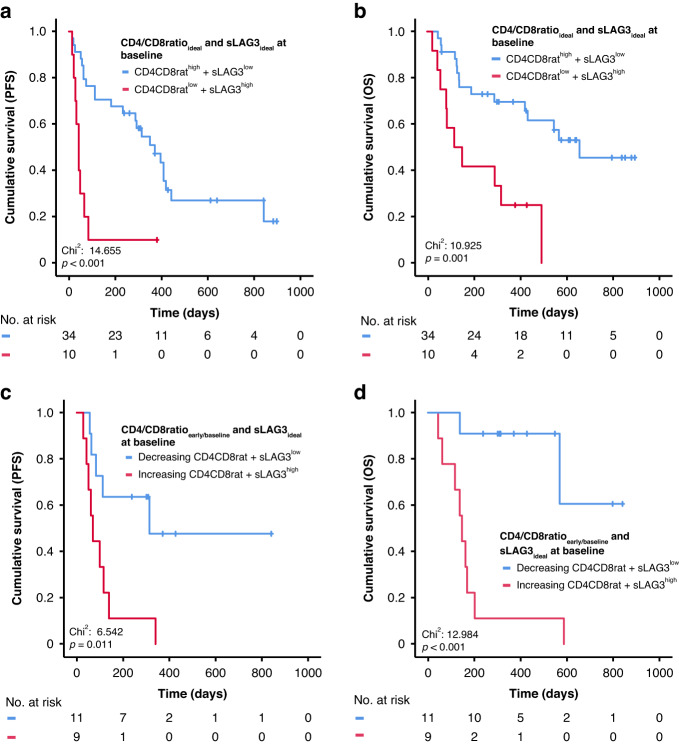
A combined immunological score comprising sLAG3 concentrations and CD4/CD8 ratio values in peripheral blood is a predictor of progression-free and overall survival in patients undergoing ICI therapy in advanced solid malignancies. Patients with a baseline CD4/CD8 ratio above the ideal cut-off (1.25) and sLAG3 levels below the ideal-cut off (31.85 pg/ml) (CD4CD8rat^high^ + sLAG3^low^) show a substantially improved progression-free (**a**) and overall survival (**b**) compared to patients with an unfavorable CD4CD8rat^low^ + sLAG3^high^ profile. During therapy, patients with decreasing CD4/CD8 ratio comparing baseline to the early-time point and sLAG3 levels below a pretreatment ideal cut-off for this subpopulation (sLAG3: 22.64 pg/ml) respond (**c**) and survive (**d**) significantly longer than patients with an increasing CD4/CD8 ratio combined with sLAG3 values above the cut-off (increasing CD4/CD8 ratio + sLAG3^high^).

We finally assessed whether a combination of sLAG3 concentrations and the CD4/CD8 ratio values as important modulators of the antitumoral response might pose as outcome predictors of patients with advanced cancer under ICI therapy. For baseline values, we considered a combined immune status score comprising the ideal cut-off of the CD4/CD8 ratio and a newly generated ideal cut-off for sLAG3 for the immune status subset of *n* = 63 (31.85 pg/ml, p_PFS_ < 0.001, p_OS_ = 0.002). This combined score showed a highly significant predictive potential that transcended the relevance of either of the parameters by themselves (p_PFS_=0.001, p_OS_ = 0.001). Patients with a beneficial CD4CD8rat^high^ + sLAG3^low^ profile had a median PFS of 371 days and OS of 658 days compared to only 42 and 117 days among patients with a CD4CD8rat^low^ + sLAG3^high^ profile (HR_PFS_: 4.592 [1.961–10.752]; *p* < 0.001, HR_OS_: 3.838 [95%CI: 1.631–9.035], *p* = 0.002, Fig. 4a, b).

As demonstrated before, the best way to use CD4/CD8 ratio as a predictive marker longitudinally is by assessing its dynamics rather than static values. This hypothesis was further sustained by the high predictive potency of a combined immunological score for patients during immunotherapy combining the ideal pre-treatment cut-off for sLAG-3 (sLAG3: 22.64 pg/ml with p_PFS_ = 0.014 and p_OS_ = 0.001, *n* = 38) and the dynamics of the CD4/CD8 ratio (decreasing/increasing). Patients with a profile comprising a decreasing CD4/CD8 ratio between baseline and the early time-point and initial sLAG3 levels below the ideal cut-off had significantly extended PFS of 314 days and not reached median OS compared to patients with an increasing ratio and initial sLAG3 levels under the cut-off value with a median PFS of 70 days and OS of 147 days (p_PFS_=0.011 and p_OS_ < 0.001, Fig. 4c, d). Cox-regression analysis confirmed the high predictive power of this combined score for PFS (HR: 3.850 [95%CI: 1.273–11.649], *p* = 0.017) and OS (HR: 10.304 [95%CI: 2.183–48.634], *p* = 0.003).

## Discussion

Since 2010, a revolution in cancer immunotherapy was started by the CTLA-4 antibody Ipilimumab [1]. An even greater breakthrough was seen with the appearance of PD-1/PDL-1 antibodies Nivolumab and Pembrolizumab [2]. Ever since, seven ICI were approved for the therapy of malignant melanoma, lung cancer, renal cell carcinoma, Hodgkin lymphoma and many other entities [3]. A wide range of drugs will follow that are being currently evaluated in more than 100 clinical trials [21], targeting new molecules including the next generation immune checkpoint LAG-3. Despite the great promise behind inhibiting different immune checkpoints, only less than a third of metastatic cancer patients experience long-term survival. The toxicities, the costs and the fact that these patients would in some cases even benefit more from conventional chemotherapy or other treatment regimens, deems the search for biomarkers to select ideal therapeutical candidates essential [22].

In this study, we found that sLAG3 and correlating circulating T-cell subsets can be used as a non-invasive predictive marker to predict outcome to ICI therapy. As such, patients with a serum concentration of sLAG3 above an ideal cut-off before ICI therapy had a median PFS of 71 days and OS of 151 days compared to 314 and 547 days if levels were below this value. In addition, the CD4/CD8 cell ratio and its dynamics during therapy as well as a combined sLAG3-CD4/CD8 ratio score were also strong predictors of OS.

LAG-3 is a co-inhibitory receptor expressed by different T-lymphocyte subsets such as activated T cells and Tregs, but also B-lymphocytes, natural killer (NK) cells and DCs. Canonically, it binds to APC derived MHC-II with a tenfold higher affinity than CD4, but some studies have suggested the interference with the MHCII-CD4 interaction is not mainly responsible for its coinhibitory function [23]. It also unfolds its T-cell suppressing function through at least three other ligands such as FGL-1, a liver secreted protein which is also highly upregulated in several cancers, Gal-3, which derives from tumor cells and activated T-lymphocytes and LSECtin, mainly produced by tumor cells [5, 24]. However, the interactions of LAG3 with its ligands and consequent signaling cascades, specially the recently discovered α-synuclein fibrils (α-syn), which seems to play a role in Parkinson’s disease [25], are not yet fully understood. Similarly to PD-(L)1 and CTLA-4, inhibitors against this membrane-bound immune checkpoint in form of mAbs are in use in clinical practice and have shown promising results in several trials. Currently, more than 15 agents targeting LAG3 are being investigated in clinical trials [26]. On the one hand, these boost antitumoral response by targeting membrane-bound LAG-3 in T cells, the most current example being the drug relatlimab, showing interesting effectivity when combined with an anti PD-1 agent in a phase III trial for metastatic melanoma [27]. On the other hand, targeting sLAG3 by mimicking its effect and agonizing MHC-II in APCs, leads to their maturation and activation [8]. Human sLAG3 is produced via alternative splicing and proteolytic cleavage of the extracellular domain and is mainly secreted by DCs but also B and T lymphocytes and its biological function remains vastly unresolved [28, 29]. It has been regarded as a mere product of membrane bound LAG3 cleavage with no biological function, but meaningful due to the consequent decreased presence of coinhibitory LAG3, enhancing T cell proliferation and effector function [6]. However, some studies have shown its own coinhibitory function through impairment of monocyte differentiation into macrophages and DCs, which themselves then have reduced immune activating function [10]. It is of note that human sLAG3, which binds to MHC-II with scarce affinity, is not equivalent to synthetic sLAG3 mimicking Ig-fusion proteins that bind with high affinity to MHC-II with consequent immune response potentiation, such as eftilagimod alpha [5]. Nevertheless, one study in gastric cancer shows how application of recombinant sLAG-3 in mice can unfold a costimulatory role by inhibiting tumor growth through promotion of CTL derived IL-12 and IFN-γ secretion [30].

The diagnostic, predictive and prognostic potential of sLAG3 has been demonstrated in some disease settings with divergent results. In active tuberculosis, high values of sLAG3 were associated with better disease control and cure [12]. In metastatic hormone-receptor positive breast cancer, progression-free survival (PFS) and OS was better for patients with detectable sLAG3 levels at diagnosis [15], while contrastingly in lung cancer, high sLAG3 levels resulted in a significantly shorter relapse free survival [31]. Within the frame of ICI based therapy, sLAG3 has only been studied in small cohorts comprising mostly a single tumor entity under therapy with a single ICI agent [16, 32]. One common trait of all the ICI based studies is that high levels of sLAG-3 predicted worsened PFS and OS.

We demonstrate how circulating sLAG3 before initiation of immune checkpoint blockade acts as a potent predictor of patient’s PFS and OS, an effect that could also be observed for patients with high levels of sLAG3 at an early and a late time point during therapy. In addition, we demonstrated significant correlations between sLAG3 and the abundance of different T cell subsets (positive: CD3 + CD8 + , negative: CD4/CD8 ratio, LAG3+ HTLs and CTLs), showing how this molecule could modulate important immune cell subsets. CD3 + CD8+ cells are essential for antitumoral immune responses through their direct cytotoxic potential [20]. Also CD3 + CD4+ cells seem to play a meaningful role in this setting [19]. Importantly, a recent study showed that a burst of activated CD8 + HLADR + T-cells in the early phases of immunotherapy was a common trait in responders with longer PFS and OS [33]. Also, concerning LAG3 expression in HTLs/CTLs, findings are divergent with high abundance of LAG3 + CD4+ and CD8+ cells being deemed as unfavorable for survival, depicting a more inhibitory immune state with less inherent T cell activation [34], whereas other studies have shown a positive correlation between high frequencies these cells and OS [35]. The interpretation of the significant negative correlation between sLAG3 and membrane bound LAG3 expression in T cells is limited by the small subset number (*n* = 6), but might indicate that most of the sLAG3 derived from proteolytic cleavage of LAG3 on the membrane of CD8+ and CD4 + T lymphocytes, lowering the levels of measurable expression on their surface in the periphery. Nevertheless, we assume sLAG3^high^ patients might have high numbers of LAG3 expressing T lymphocytes in the tumor microenvironment, a known mechanism of evasion to anti PD-1 therapy [36].

Due to the relevance of both CD4+ and CD8 + T cell quantification in ICI response [37, 38], we assessed and depicted the impact of the CD4/CD8 ratio in the peripheral blood in the outcome of our patients. Patients with a high CD4/CD8 ratio, meaning a physiological higher abundance of CD4+ related to CD8 + T cells in the periphery, at baseline and during therapy, responded better to therapy and lived longer. The most noteworthy findings were shown when evaluating the dynamics of the CD4/CD8 ratio, demonstrating that patients with a decreasing ratio, ergo a shift towards higher abundance of CD8 + T cells, live substantially longer, mainly when the effect is evaluated using baseline values compared to values at an early time-point of ICI therapy, after one or two cycles (*p* = 0.012, HR = 3.324 for ∆ baseline/early time-point), possibly denoting the importance of early recruitment of CD8 + T cells that can effectively combat the tumor as an immediate result of ICI based immune boost, compatible with prior findings [33, 39]. The relevance of a higher CD4/CD8 ratio with a most significant cut-off nearing established laboratorial reference values (ideal cut off: 1.25, laboratorial reference value > 1), might point towards how a healthy distribution of T cell subsets might indicate a healthier and more capable immune system prone to successful activation by immune checkpoint inhibitors. In addition, different studies have shown the fundamental role of CD4+ cells in antitumor immunity, who ultimately steer the direct cytotoxic response by CD8+ cells [40]. In line, reversed CD4/CD8 ratios have been associated with an impaired immune function, relevant in autoimmune disease [41] and cancer [42, 43]. We hypothesize that patients with a reversed CD4/CD8 ratio in our cohort lack a balanced distribution of T cell subsets with a needed predominance of T helper cells (CD3+ CD4 +), which upon successful activation by ICI can, via necessary CD4+ mediation, manage a higher recruitment of CD8+ cells (achieving ultimately a prognostically favorable decrease in the CD4/8 ratio), that convey the direct cytotoxic response against cancer. Considering these findings, we established a prognostically highly relevant immunological score comprising values of CD4/CD8 ratio and sLAG3 concentrations in serum, which outweighs the prediction capacity of any of these values by themselves. At baseline, a combination of ideal cut-offs for the CD4/CD8 ratio and sLAG3 has a meaningful predictive impact (p_PFS_ = 0.001, HR_PFS_:4.592, p_OS_ = 0.001, HR_OS_: 3.838), with patients bearing an ideal profile consisting of CD4/CD8ratio^high^ + sLAG3^low^ responding and living substantially longer (PFS: 371 vs 42 days, OS: 658 vs. 117 days in the negative profile of CD4/CD8ratio^low^ + sLAG3^high^). For longitudinal analyses, CD4/CD8 ratio dynamics (increasing/non-increasing) combined with the ideal sLAG3 pretreatment cut-off act as a highly relevant therapeutic monitoring score. For the early-time point, patients with an unfavorable combination of increasing CD4/CD8 ratio and a sLAG3 value above the ideal cut-off were at an impressive 10.3-times higher death risk (and 3.9-times higher progression risk) than patients with favorable profile.

Our limitations are mainly based on the heterogeneity of our patient cohort, composed by several tumor entities undergoing therapy with different ICI agents. However, result interpretation might be facilitated by the single center longitudinal design that conveys a solid comparability concerning different laboratorial parameters, demographic attributes and different treatment time points. Furthermore, the lack of other therapies such as chemotherapy or radiotherapy leaves the question, whether these values have a unique relevance in ICI therapy or can also be employed in different therapeutic modalities, unanswered. Due to the small sample size for each tumor entity, an analysis corroborating the predictive value of the biomarkers within each entity would not be accurate in the case of our cohort. For the negative correlation between sLAG3 concentrations in serum and expression of LAG3 in CD3+ CD8+ or CD3+ CD4+ cells, we deliver encouraging results, but it is based on a small patient cohort (*n* = 6) and warrants confirmation in larger patient collectives, even with approaches that also take the TME and tumor infiltrating-lymphocytes (TILs) into consideration for better mechanistic comprehension. To sum up, confirmatory multicenter approaches, including higher volumes of patients for each tumor entity and ICI agent, possibly combining even more immunological components of the cancer patient network (CPN), therapeutic modalities and mechanistic approaches are warranted, so that sLAG3, CD4/CD8 ratio and their combined immunological score can be assessed to gain deeper knowledge into the predictive role of these molecules.

To sum up, considering the known role of sLAG3 as a coinhibitory immune modulator leading to monocyte differentiation impairment, our results corroborate how this molecule seems to impede an effective antitumoral immune response for patients before and during immune checkpoint blockade. Consequently, sLAG3 seems to constitute a feasible and highly relevant predictive biomarker in this setting. When combined with the CD4/CD8 ratio, whose higher static values before therapy express a well-regulated and balanced immune status prone to successful stimulation with ICI and whose decreasing dynamic values after initiation of immunotherapy might indicate a wanted shift towards a higher abundance of CD8+ cells, which are vital for direct cytotoxic antitumoral response, an unprecedented and highly potent predictive score to ICI therapy can be established. A combination score of biomarkers depicting the immune status tends to be more successful in depicting response to ICI therapy, since single values are prone to high fluctuance due to the complex interplay of several immunomodulatory components in the CPN.

### Supplementary information

REMARK Checklist BJC sLAG3 ICI Manuscript

Supplementary Material sLAG3 ICI Manuscript

## Data Availability

Data will be made available, upon reasonable request, by the corresponding author.
