# Supplementary material for: Soluble lymphocyte activation gene-3 (sLAG3) and CD4/CD8 ratio dynamics as predictive biomarkers in patients undergoing immune checkpoint blockade for solid malignancies
Source: Br J Cancer. 2024 Jan 17;130(6):1013–22. doi: 10.1038/s41416-023-02558-7 (PMC10951205; doi:10.1038/s41416-023-02558-7)

Soluble lymphocyte activation gene-3 (sLAG3) and CD4/CD8 ratio dynamics as predictive biomarkers in patients undergoing immune checkpoint blockade for solid malignancies

Joao Gorgulho[1,2], Christoph Roderburg[3,4], Fabian Beier[4,5], Carsten Bokemeyer[1], Tim H. Brümmendorf[4,5], Sven H. Loosen[3,4,#], Tom Luedde[3,4,#]

[1] Department of Oncology, Hematology and Bone Marrow Transplantation with Section of Pneumology, University Medical Centre Hamburg-Eppendorf, Martinistraße 52, 20251 Hamburg, Germany.

[2] Mildred Scheel Cancer Career Center, University Cancer Center Hamburg, University Medical Center Hamburg-Eppendorf, Hamburg, Germany

[3] Department of Gastroenterology, Hepatology and Infectious Diseases, University Hospital Düsseldorf, Medical Faculty of Heinrich Heine University Düsseldorf, 40225 Düsseldorf, Germany

[4] Center for Integrated Oncology Aachen-Bonn-Cologne-Düsseldorf (CIO[ABCD]), Germany

[5] Department of Medicine IV, University Hospital RWTH Aachen, Pauwelsstrasse 30, 52074 Aachen, Germany

[#] These authors share last authorship

Table of Contents

Supplementary Tables:

- Supplementary Table 1 (page 2)
- Supplementary Table 2 (page 3)
- Supplementary Table 3 (page 4)

Supplementary Figures

- Supplementary Figure legends (page 4)
- Supplementary Figure 1 (page 5)
- Supplementary Figure 2 (page 6)
- Supplementary Figure 3 (page 7)
- Supplementary Figure 4 (page 8)
- Supplementary Figure 5 (page 9)

Supplementary Table 1. Patient characteristics of the LAG-3 T cell expression cohort.

Sample	Gender	Tumor entity	Age (years)	UICC Stage	Therapy	Prior lines systemic treatment	PDL-1 Score (%)	PFS (days)	OS (days)
Pat 1	female	NSCLC	67	III	pembrolizumab	1	80	427*	427**
Pat 2	female	melanoma	47	III	nivolumab	1	unknown	238*	238**
Pat 3	male	NSCLC	59	IV	nivolumab	1	0	83	313**
Pat 4	male	NSCLC	81	IV	pembrolizumab	0	50	301*	301**
Pat 5	female	head and neck	67	IV	nivolumab	2	unknown	42	58
Pat 6	male	urogenital tract	66	IV	nivolumab	1	unknown	70	137

NSCLC: non-small cell lung cancer, UICC: Union for International Cancer control, PFS: progression-free survival, OS: overall survival, *still responding, ** still alive

Supplementary Table 2: Serum levels of laboratory markers and peripheral T cell subsets.

	**ICI patients** median [range]
sLAG3 baseline [pg/ml]	20.1 [0.0-272.14]
sLAG3 early time point [pg/ml]	29.8 [0.67-382.53]
sLAG3 late time point [pg/ml]	25.8 [0.67-199.23]
Haemoglobin [g/l]	11.95 [3.32-17.60]
Platelets [cells/nl]	243.0 [20.0-693.0]
Leucocyte count [cells/nl]	7.1 [3.1-29.1]
Frequency Lymphocytes [%]	15.75 [2.2-62.1]
Lymphocyte count [cells/µl]	1056.8 [311.0-6409.0]
Sodium [mmol/l]	139.0 [124.0-144.0]
Potassium [mmol/l]	4.40 [3.30-6.30]
Bilirubin [mg/dl]	0.35 [0.10-3.7]
AST [U/l]	25.0 [10.0-187.0]
ALT [U/l]	18.0 [7.0-179.0]
ALP [U/l]	90.0 [35.0-1439.0]
GGT [U/l]	44.0 [9.0-1591.0]
LDH [U/l]	219.0 [8.2-1273.0]
Creatinine [mg/dl]	0.87 [0.37-6.09]
Frequency CD3+ cells [%]	71.45 [14.60-93.30]
Absolute count CD3+ cells [cells/µl]	881.0 [185.0-2745.0]
Frequency CD3+CD4+ cells [%]	41.55 [9.80-70.30]
Absolute count CD3+CD4+ cells [cells/µl]	347.5 [53.0-1090.0]
Frequency CD3+CD8+ cells [%]	21.90 [3.60-53.70]
Absolute count CD3+CD8+ cells [cells/µl]	199.5 [19.0-1375.0]
CD4+/CD8+ cell ratio baseline	1.75 [0.20-7.80]
CD4+/CD8+ cell ratio early time point	1.60 [0.10-6.10]
CD4+/CD8+ cell ratio late time point	1.90 [0.40-6.00]

sLAG3: soluble lymphocyte activation gene-3, AST: aspartate transaminase, ALT: alanine transaminase, GGT: γ-Glutamyl transpeptidase, ALP: alkaline phosphatase, LDH: lactate dehydrogenase

Supplementary Table 3. Flow cytometry data on LAG3 expression on cytotoxic and helper T lymphocytes in peripheral blood (CD3+CD4-CD8+LAG3+ and CD3+CD4+CD8-LAG3+).

Color	Sample	sLAG3 concentration	Freq. of parent CD3+CD8+LAG3+	Cell count CD3+CD8+LAG3+	MFI CD3+CD8+LAG3+	Freq. of parent CD3+CD4+LAG3+	MFI CD3+CD4+LAG3+	Cell count CD3+CD4+LAG3+
	Pat 1	5.95 pg/ml	15.5%	70/µl	422	11.4%	354	74/µl
	Pat 2	0 pg/ml	8.53%	24/µl	391	13.0%	375	100//µl
	Pat 3	6.18 pg/ml	2.86%	44/µl	323	4.43%	353	53/µl
	Pat 4	19.63 pg/ml	2.70%	16/µl	350	3.22%	344	28/µl
	Pat 5	151.18 pg/ml	1.05%	3//µl	371	2.07%	346	7/µl
	Pat 6	43.24 pg/ml	0.97%	2/µl	344	1.15%	350	4/µl

LAG3: lymphocyte activation gene 3; MFI: mean fluorescence intensity

Supplementary figure legends

Supplementary Figure 1.

Serum concentrations of sLAG3 at baseline do not significantly differ between patients with different tumor entities (A), sex (B), ECOG PS (D), smoking status (E) as well as ICI regimen (F), line of therapy (G) and side effects (H and I), but significantly differ regarding tumor stage (C).

Supplementary Figure 2.

(A) Serum sLAG3 concentrations do not significantly change throughout ICI therapy (error bars indicate standard error of the mean (SEM)). (B and C) There is no significant difference in OS between patients who show increasing or decreasing circulating sLAG3 levels at the early (C) or late time point (B) compared to baseline levels.

Supplementary Figure 3.

In a subset of patients (n=63), sLAG3 concentrations in serum before therapy initiation positively correlate with frequency and absolute count of CD3+CD8+ (CTLs) cells (A and B) and negatively correlate with the CD4/CD8 ratio (C).

Supplementary Figure 4.

(A) Flow cytometry analysis of LAG3 expression on CD3+CD8+ positive cells (CTLs) in 6 patients, the black dotted line showing the cut-off for LAG+ cells. (B and C) Comparison between lowest (B) and highest (C) LAG3 expression in CTLs. (D and E) Correlation plot of baseline sLAG3 serum levels and absolute count (D) and frequency (E) CTLs.

Supplementary Figure 5.

(A) Analysis of LAG3 expression on CD3+CD4+ positive cells (HTLs) in 6 patients, the black dotted line showing the cut-off for LAG+ cells (B) Comparison between lowest (Patient 2) and highest (Patient 6) LAG3 expression in HTLs. (C and D) Correlation plot of baseline sLAG3 serum levels and absolute count (C) and frequency (D) HTLs.

Supp. Figure 1

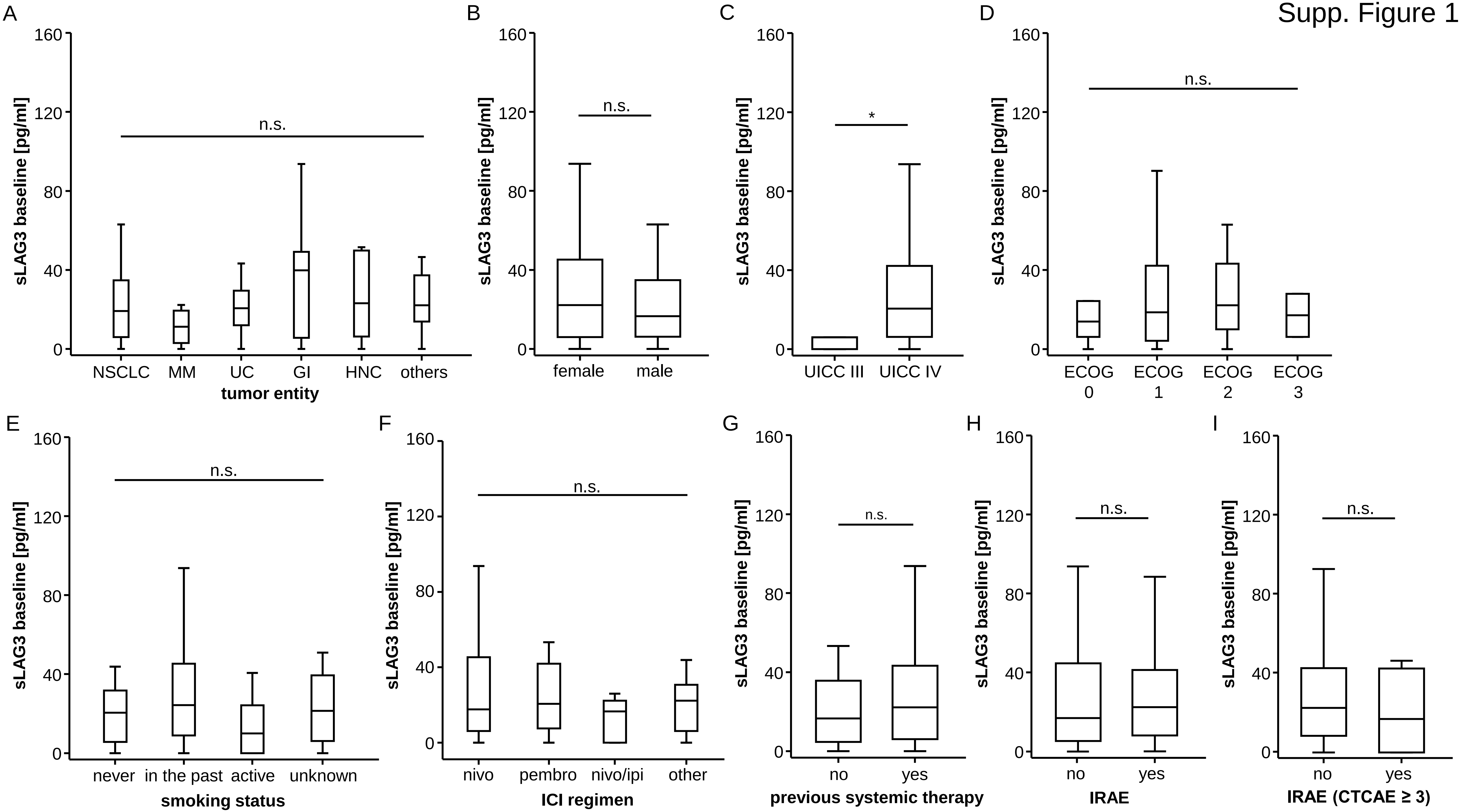

Supp. Figure 2

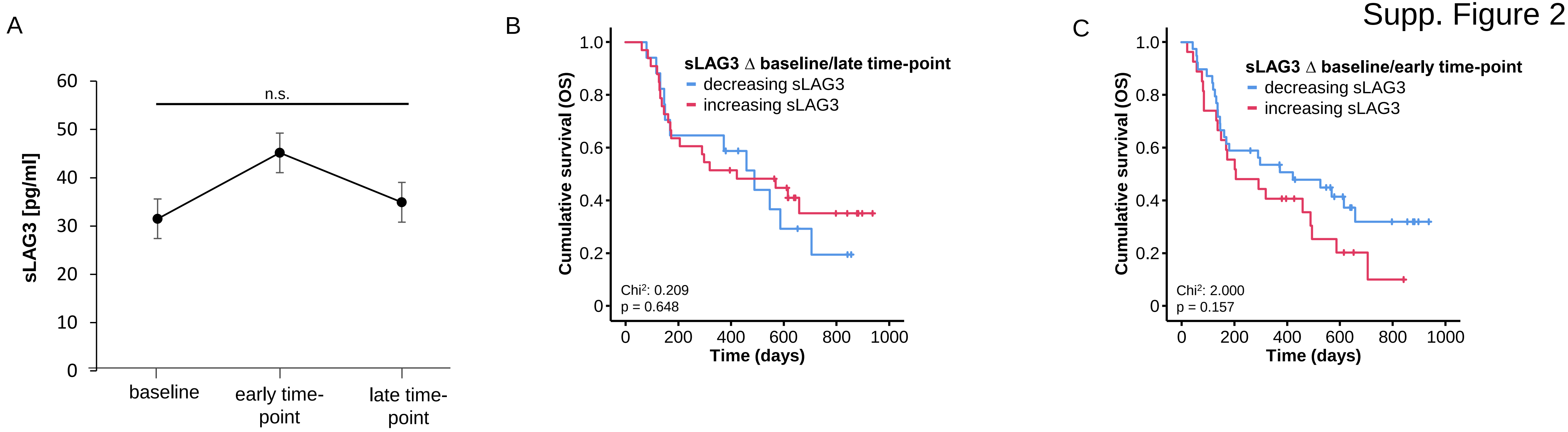

Supp. Figure 3

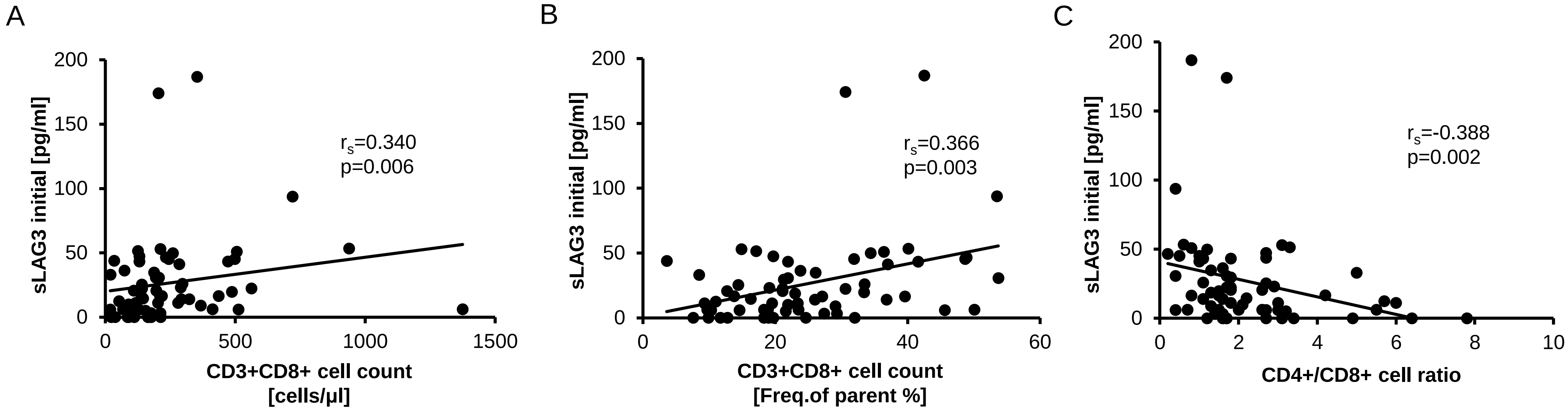

Supp. Figure 4

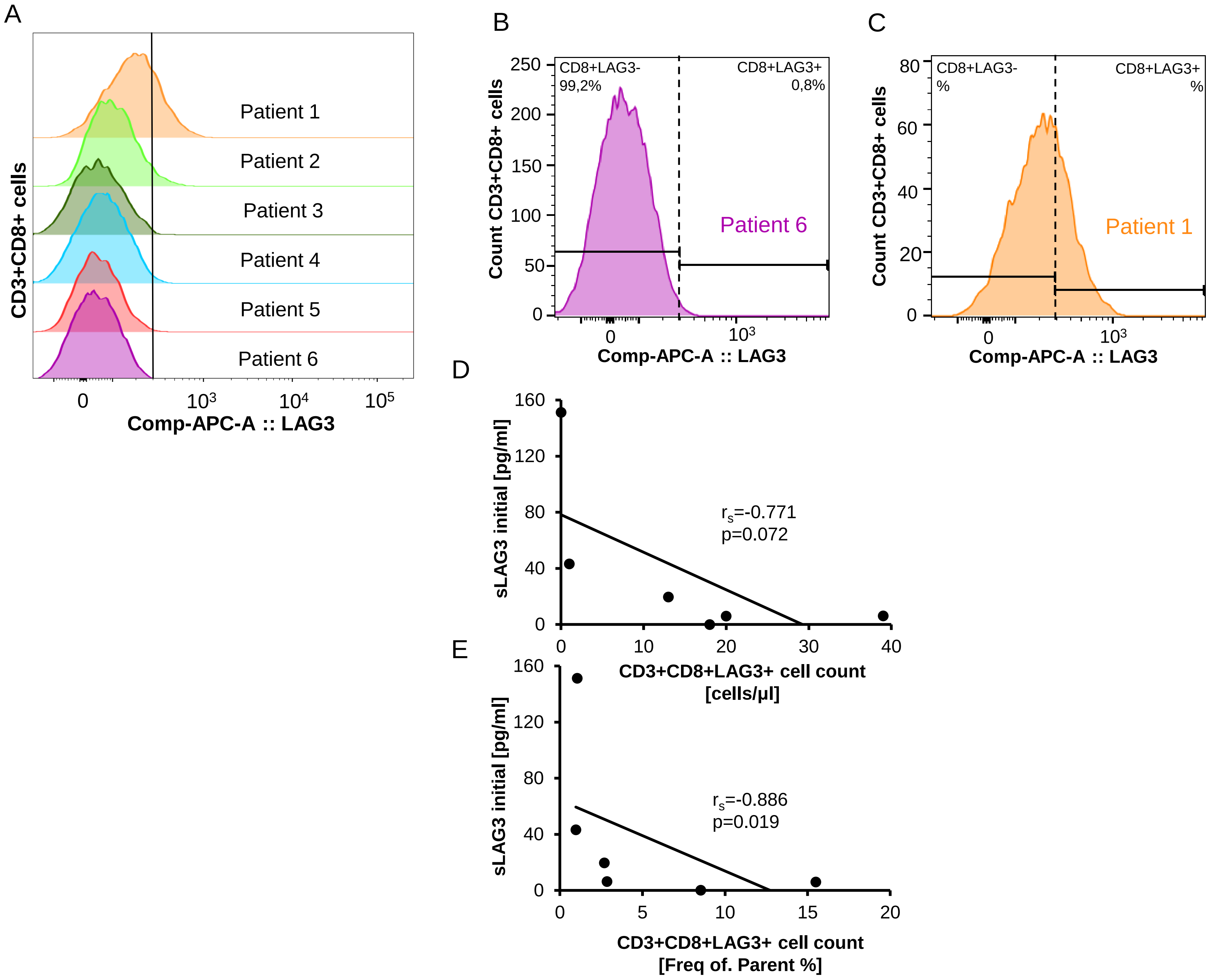

Supp. Figure 5

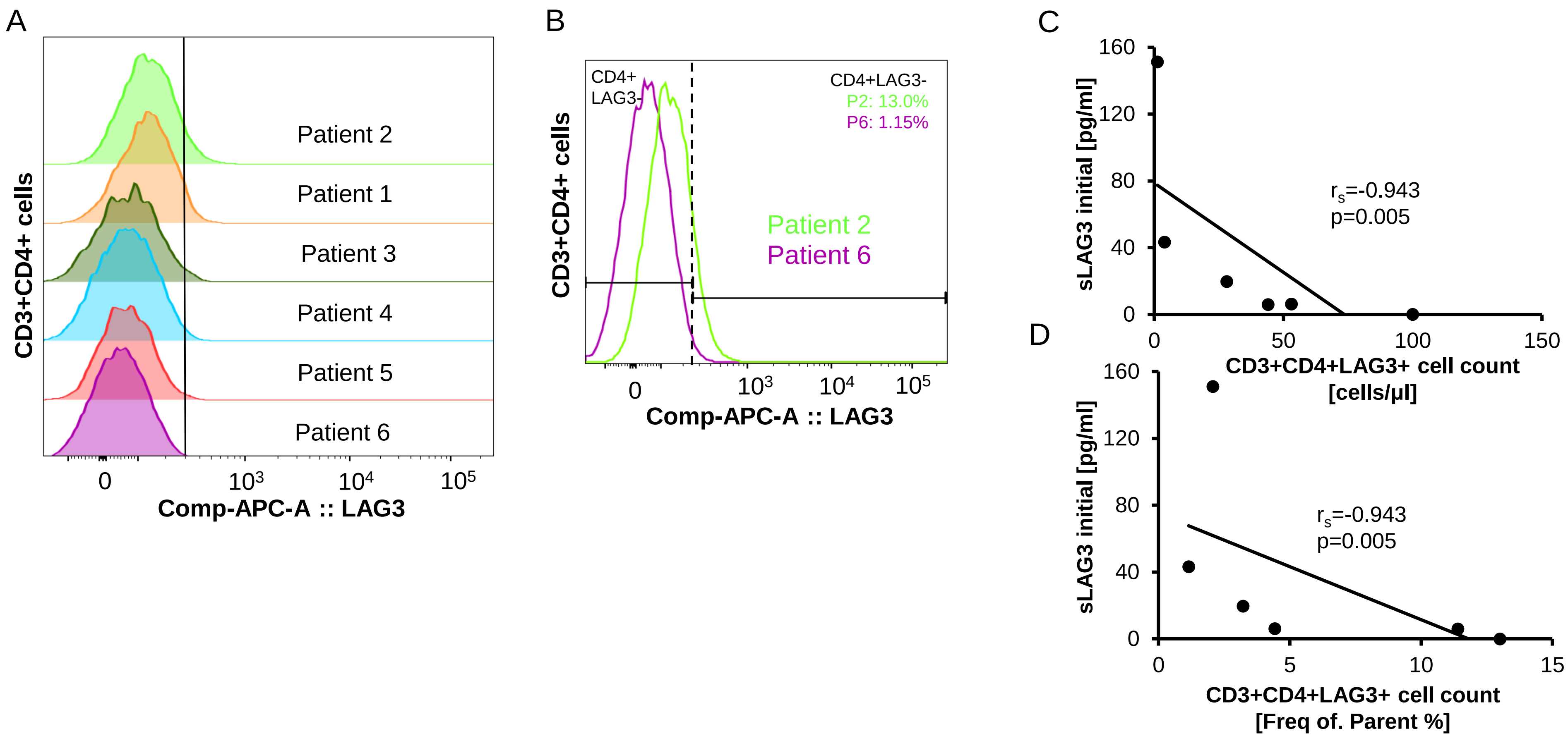

Supplement: Supplementary file 2 — Supplementary Material sLAG3 ICI Manuscript [file 41416_2023_2558_MOESM2_ESM.pdf]
